# Defining Kawasaki disease and pediatric inflammatory multisystem syndrome-temporally associated to SARS-CoV-2 infection during SARS-CoV-2 epidemic in Italy: results from a national, multicenter survey

**DOI:** 10.1186/s12969-021-00511-7

**Published:** 2021-03-16

**Authors:** Marco Cattalini, Sara Della Paolera, Fiammetta Zunica, Claudia Bracaglia, Manuela Giangreco, Lucio Verdoni, Antonella Meini, Rita Sottile, Roberta Caorsi, Gianvincenzo Zuccotti, Marianna Fabi, Davide Montin, Alessandra Meneghel, Alessandro Consolaro, Rosa Maria Dellepiane, Maria Cristina Maggio, Francesco La Torre, Alessandra Marchesi, Gabriele Simonini, Alberto Villani, Rolando Cimaz, Angelo Ravelli, Andrea Taddio, on behalf of the Rheumatology Study Group of the Italian Pediatric Society, Paolo Adamoli, Maria Concetta Alberelli, Clotilde Alizzi, Patrizia Barone, Veronica Bennato, Francesca Biscaro, Grazia Bossi, Andrea Campana, Maurizio Carone, Adele Civino, Giovanni Conti, Eleonora Dei Rossi, Emanuela Del Giudice, Alice Dell’Anna, Maia De Luca, Enrico Felici, Giovanni Filocamo, Ilenia Floretta, Maria Loreta Foschini, Marcello Lanari, Bianca Lattanzi, Alessandra Lazzerotti, Francesco Licciardi, Alessandra Manerba, Savina Mannarino, Achille Marino, Agostina Marolda, Laura Martelli, Giorgia Martini, Angela Mauro, Maria Vincenza Mastrolia, Angelo Mazza, Angela Miniaci, Francesca Minoia, Alma Olivieri, Guido Pennoni, Rossana Pignataro, Francesca Ricci, Donato Rigante, Matilde Rossi, Claudia Santagati, Martina Soliani, Silvia Sonego, Domenico Sperlì, Sara Stucchi, Barbara Teruzzi, Elpidio Tierno, Tatiana Utytatnikova, Piero Valentini, Gianluca Vergine

**Affiliations:** 1 Pediatrics Clinic, ASST Spedali Civili di Brescia, University of Brescia, Piazzale Spedali Civili 1, 25123 Brescia, Italy; 2 University of Trieste, Piazzale Europa, 2 Trieste, Italy; 3 Division of Rheumatology, Bambino Gesù Children’s Hospital, IRCCS, Pizza di Sant’Onofrio, 4, 00165 Rome, Italy; 4 Institute for Maternal and Child Health, IRCCS “Burlo Garofolo”, Via dell’Istria 65/1, 34137 Trieste, Italy; 5 Paediatric Department, Hospital Papa Giovanni XXIII, Piazza OMS 1, 24127 Bergamo, Italy; 6 Department of Paediatrics, Pediatria 2, Santobono-Pausilipon Children’s Hospital, Via Mario Fiore 6, 80129 Naples, Italy; 7 UOSD Centro Malattie Autoinfiammatorie ed Immunodeficienze, IRCCS Istituto Giannina Gaslini, Via Gerolamo Gaslini 5, 16147 Genoa, Italy; 8 Department of Pediatrics, University of Milan, Children’s Hospital V Buzzi, Via Lodovico Castelvetro 32, 20154 Milan, Italy; 9 Department of Pediatrics, University of Bologna, IRCCS Sant’Orsola-Malpighi Hospital, Via Giuseppe Masserenti 9, 40138 Bologna, Italy; 10 Department of Pediatrics and Public Health, University of Turin, Via Giuseppe Verdi 8, 10124 Turin, Italy; 11 Department of Woman’s and Child’s Health, University of Padova, Via 8 Febbraio 1848, 35122 Padua, Italy; 12 Clinica Pediatrica e Reumatologia, IRCCS Istituto Giannina Gaslini and DINOGMI, Università di Genova, Via Gerolamo Gaslini 5, 16147 Genoa, Italy; 13 Pediatric Intermediate Care Unit, Fondazione IRCCS Ca’ Granda Ospedale Maggiore Policlinico, Via della Commenda 9, 20122 Milan, Italy; 14 Department of Health Promotion Sciences Maternal and Infantile Care, Internal Medicine and Medical Specialities “G. D’Alessandro”, University of Palermo, Via del Vespro 133, 90127 Palermo, Italy; 15 Pediatric Rheumatology Center, Pediatric Unit, “Giovanni XXIII”, Pediatric Hospital, Via Giovanni Amendola 207, 70126 Bari, Italy; 16 Bambino Gesu’ Children’s Hospital, IRCCS, Piazza Sant’Onofrio 4, 00165 Rome, Italy; 17 Pediatric Rheumatology Unit, AOU Meyer, University of Florence, Via Gaetano Pieraccini 24, 50139 Florence, Italy; 18 Department of Clinical Sciences and Community Health, University of Milan, Via Commenda 19, 20122 Milan, Italy

**Keywords:** SARS-CoV-2, Kawasaki disease, Pediatric inflammatory multisystem syndrome-temporally associated to SARS-CoV-2 infection, Myocarditis, Hypotension, Multisystem inflammatory syndrome associated with coronavirus disease, Coronary artery abnormalities

## Abstract

**Background:**

There is mounting evidence on the existence of a Pediatric Inflammatory Multisystem Syndrome-temporally associated to SARS-CoV-2 infection (PIMS-TS), sharing similarities with Kawasaki Disease (KD). The main outcome of the study were to better characterize the clinical features and the treatment response of PIMS-TS and to explore its relationship with KD determining whether KD and PIMS are two distinct entities.

**Methods:**

The Rheumatology Study Group of the Italian Pediatric Society launched a survey to enroll patients diagnosed with KD (Kawasaki Disease Group – KDG) or KD-like (Kawacovid Group - KCG) disease between February 1st 2020, and May 31st 2020. Demographic, clinical, laboratory data, treatment information, and patients’ outcome were collected in an online anonymized database (RedCAP®). Relationship between clinical presentation and SARS-CoV-2 infection was also taken into account. Moreover, clinical characteristics of KDG during SARS-CoV-2 epidemic (KDG-CoV2) were compared to Kawasaki Disease patients (KDG-Historical) seen in three different Italian tertiary pediatric hospitals (Institute for Maternal and Child Health, IRCCS “Burlo Garofolo”, Trieste; AOU Meyer, Florence; IRCCS Istituto Giannina Gaslini, Genoa) from January 1st 2000 to December 31st 2019. Chi square test or exact Fisher test and non-parametric Wilcoxon Mann-Whitney test were used to study differences between two groups.

**Results:**

One-hundred-forty-nine cases were enrolled, (96 KDG and 53 KCG). KCG children were significantly older and presented more frequently from gastrointestinal and respiratory involvement. Cardiac involvement was more common in KCG, with 60,4% of patients with myocarditis. 37,8% of patients among KCG presented hypotension/non-cardiogenic shock. Coronary artery abnormalities (CAA) were more common in the KDG. The risk of ICU admission were higher in KCG. Lymphopenia, higher CRP levels, elevated ferritin and troponin-T characterized KCG. KDG received more frequently immunoglobulins (IVIG) and acetylsalicylic acid (ASA) (81,3% vs 66%; *p* = 0.04 and 71,9% vs 43,4%; *p* = 0.001 respectively) as KCG more often received glucocorticoids (56,6% vs 14,6%; *p* < 0.0001). SARS-CoV-2 assay more often resulted positive in KCG than in KDG (75,5% vs 20%; p < 0.0001). Short-term follow data showed minor complications. Comparing KDG with a KD-Historical Italian cohort (598 patients), no statistical difference was found in terms of clinical manifestations and laboratory data.

**Conclusion:**

Our study suggests that SARS-CoV-2 infection might determine two distinct inflammatory diseases in children: KD and PIMS-TS. Older age at onset and clinical peculiarities like the occurrence of myocarditis characterize this multi-inflammatory syndrome. Our patients had an optimal response to treatments and a good outcome, with few complications and no deaths.

**Supplementary Information:**

The online version contains supplementary material available at 10.1186/s12969-021-00511-7.

## Background

Italy was the first Western Country to be hit by the SARS-CoV-2 epidemic. To date, more than 236,000 cases have been diagnosed, with more than 32,000 deaths. Children accounted for almost 2% of infections, with an estimated mortality rate of 0,2% [1]. These data confirm previous reports on lower rates of SARS-CoV-2 infection and milder forms of the disease in children, compared to adults [2–4]. Nonetheless, few weeks after the epidemic peak, an abnormally high number of severely ill children were seen in those areas of the country with higher SARS-CoV-2 incidence [5–7]; these observations were then confirmed in other European countries [8–10]. There is now mounting evidence on the existence of a childhood multi-inflammatory syndrome related to SARS-CoV-2 infection sharing some similarities with Kawasaki Disease (KD) and Toxic Shock Syndrome (TSS) [9]. This condition has been named such as Pediatric Inflammatory Multisystem Syndrome-temporally associated to SARS-CoV-2 infection (PIMS-TS) or Multisystem Inflammatory Syndrome associated with Coronavirus Disease 2019 (MIS-C) [11, 12].

However, the real extent of the clinical spectrum of disease, and the exact role of SARS-CoV-2 infection, are still poorly understood. Moreover, it is still not clear if SARS-CoV-2 might also be considered a trigger for KD development or if KD, during the SARS-CoV-2 epidemic, presented peculiar and unusual clinical manifestations.

Our study aimed to build a national survey for patients with KD or KD-like multisystemic disease during SARS-CoV-2 epidemic evaluating clinical manifestations, laboratory data, treatment, outcome, and relationship with virus outbreak.

## Methods

### Study design and patient selection

This is an observational, retrospective, multicenter study. Institutional Review Board approval was achieved (IRCCS Burlo Garofolo-03/2020). The Rheumatology Study Group of the Italian Pediatric Society launched a national, online, survey on April 24th, 2020 to enroll those patients diagnosed with KD or KD-like multisystemic disease during SARS-CoV-2 epidemic. The children hospitalized between February 1st 2020, and May 31st 2020 with the clinical diagnosis of classical or incomplete-KD (iKD) as well as KD-like multi-inflammatory syndrome were enrolled.

The clinical classification was: 1) KD and iKD diagnosis, named as Kawasaki Disease Group (KDG), based on the fulfilment of the American Heart Association criteria [13]; 2) KD-like multi-inflammatory syndrome diagnosis, named as KawaCOVID Group (KCG), based on the presence of i) persistent fever (> 48 h), lymphopaenia and evidence of single or multi-organ dysfunction with other additional clinical, laboratory or imagining; ii) exclusion of any other microbial cause [11].

In consideration of the retrospective nature of the study, an expert panel of pediatric rheumatologists (AR, GS, CB, MC, RC), blinded for patients’ recruiting center, was asked to review every patient included in the database to check the correct patient classification or eventually reclassify them properly. Those patients who did not fulfill any of the above-mentioned criteria were excluded from the study.

Since SARS-CoV-2 spread with different prevalence through Italy, with Piedmont and Lombardy being the most heavily hit regions, we also compared the data of patients from these two regions with the data of patients from the other regions irrespectively from diagnostic classification.

At the end, clinical characteristics of KDG during SARS-CoV-2 epidemic were compared to Kawasaki Disease patients seen in three different Italian tertiary pediatric hospitals (Institute for Maternal and Child Health, IRCCS “Burlo Garofolo”, Trieste; AOU Meyer, Florence; IRCCS Istituto Giannina Gaslini, Genoa) from January 1st 2000 to December 31st 2019.

### Data collection

Demographic, clinical, and laboratory data, treatment information, and patient outcome were collected in an online anonymized database which was built for the study purpose (RedCAP®). Data about complications and last follow-up after discharge were collected where available. Results from all tests for SARS-CoV-2 infection with an RT-PCR assay and/or with a serologic assay were also reported. Clinical data collected were part of the normal standard of care.

### Statistical analysis

Categorical variables are described as absolute frequency and percentage while continuous variables as median and interquartile range. Chi square test or exact Fisher test were applied to evaluate the association between two categorical variables while non parametric Wilcoxon Mann-Whitney test was used to study differences between two groups of a categorical variable on a continuous variable. A *p*-value < 0·05 was considered as statistically significant. Statistical analysis was conducted using SAS software, Version 9·4 (SAS Institute Inc., Cary, NC, USA).

## Results

**Fig. 1:**
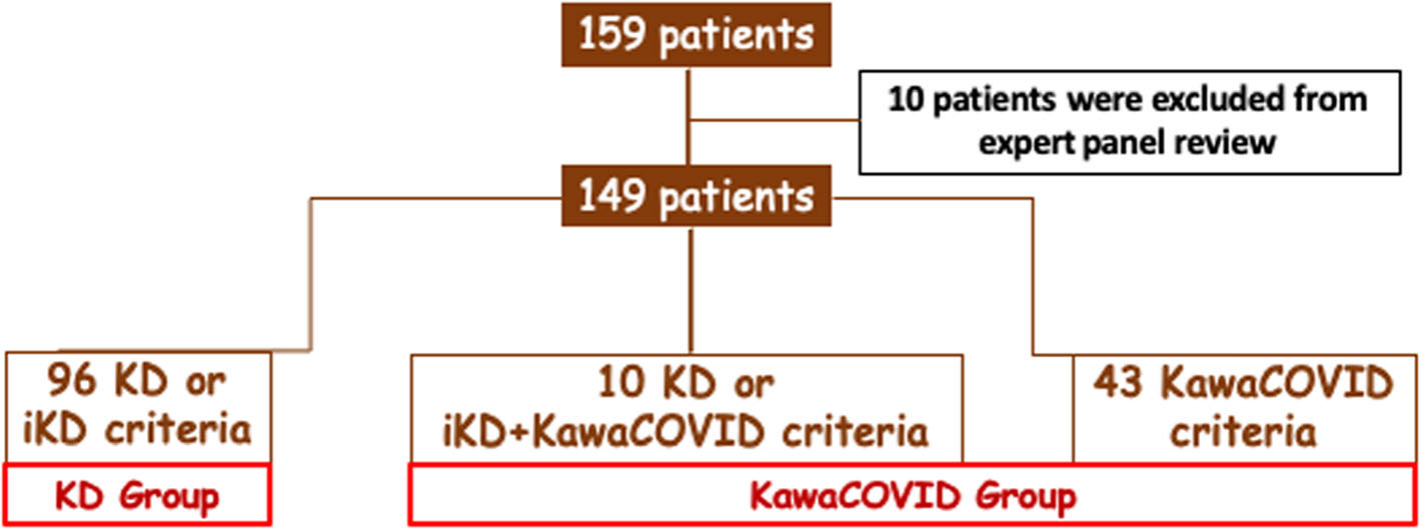
Enrollment of patients flow chart. One hundred fifty-nine patients were initially enrolled; 10 were then excluded form expert panel revision. Ninety-six patients fitted KD or iKD diagnosis. Ten patients fitted both KD/iKD or Kawacovid criteria while 43 patients filled Kawacovid criteria. KD=Kawasaki Disease; iKD = incomplete Kawasaki Disease

Emails to more than 10,000 members of the Italian Pediatric Society were sent. Data from 194 patients were entered into the database. After reviewing all the data for duplicates and files with missing information 159 cases were sent to the case definition committee. Ten patients were excluded by the committee since they didn’t fulfill the inclusion criteria. One hundred forty-nine patients with a final diagnosis of KDG or KCG were included in the study. Sixty-nine patients satisfied KD criteria, 37 iKD criteria, and 43 KawaCOVID criteria. Among the 37 patients classified as iKD, 10 satisfied also KawaCOVID criteria and were then associated to the last group (Fig. 1). The population consisted of 84 males and 65 females, the median age at the time of diagnosis was 3 years (IQR: 1–6 years). Sixty-four out of 149 patients reported at least one symptom in the 3 months before hospital admittance suggestive for SARS-CoV-2 infection; only one patient (with diarrhea and close contact to a confirmed SARS-CoV-2 patient) had a positive PCR nasal swab for SARS-CoV-2 1 month before admission, while 29 patients were tested by nasal swab before disease onset for close contact with confirmed cases, but resulted negative.

**Fig. 2:**
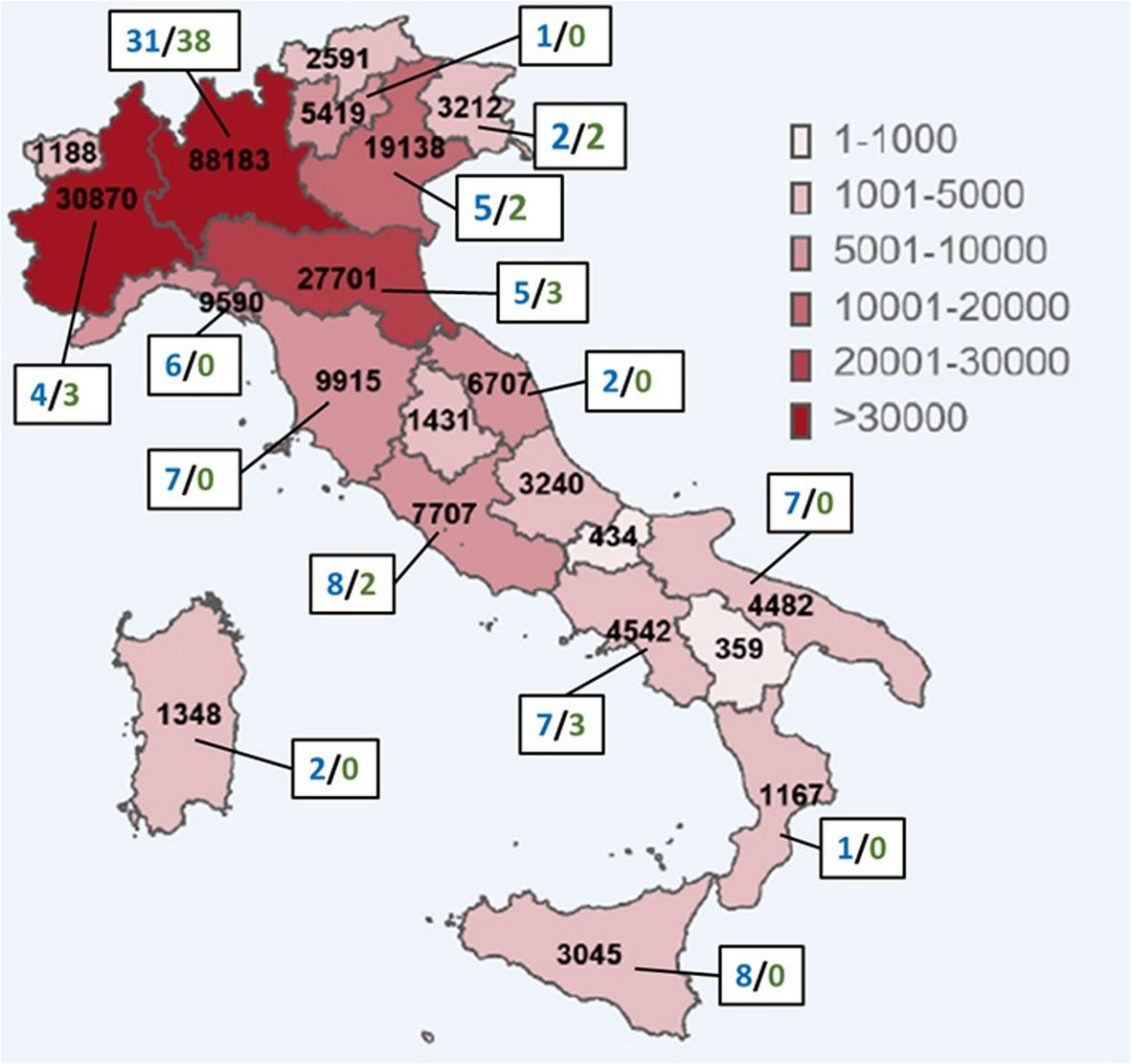
SARS-CoV-2 infection and KD/KawaCOVID patients in Italy. Distribution of KDG patients (blue numbers) and KCG (green numbers) in different areas per SARS-CoV-2 incidence (right squares indicate SARS-CoV-2 number of patients/general population). KDG = Kawasaki Disease Group; KCG = KawaCOVID Group

More than half of the patients (76/149) were from highly endemic regions (≥30,000 SARS-CoV-2 confirmed cases in the general population), while the others (73/149) came from regions with moderate-low numbers of SARS-CoV-2 confirmed cases (< 30,000 cases in the general population). Distribution of KDG and KCG cases per endemic areas is represented in Fig. 2.

### KDG vs KCG at time of diagnosis

**Fig. 3:**
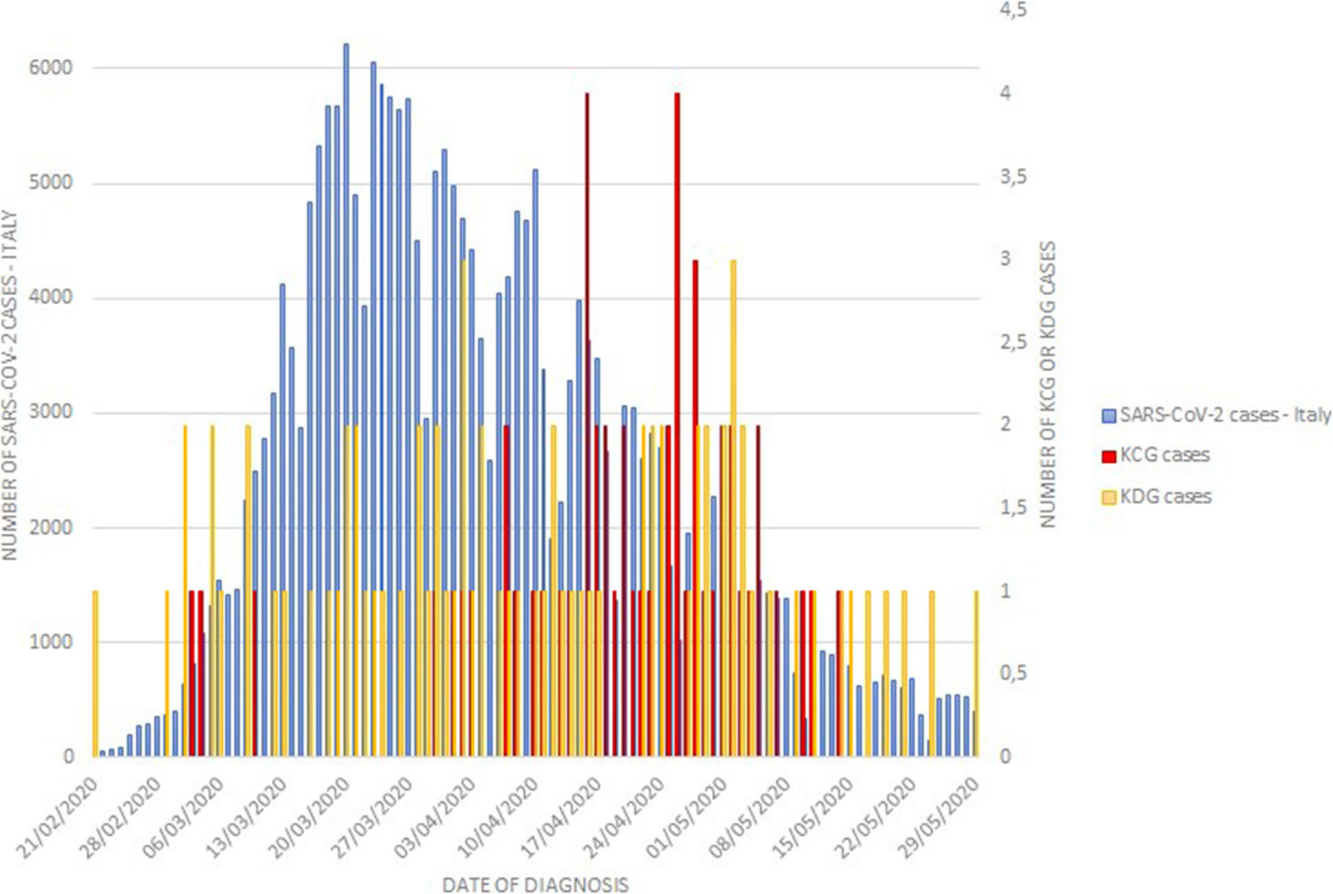
Trend of clinical manifestations among the KawaCOVID Group (KCG) and the Kawasaki Disease Group (KDG)

KCG children were significantly older (7 years; IQR: 4,5–11 years vs 2 years; IQR: 1–4 years; *p* < 0,0001). Skin and mucosal involvement were more frequent among KDG, reaching statistical significance. Moreover, the presence of conjunctivitis was more frequent among KDG as well as the evidence of irritability. On the other hand, gastrointestinal and respiratory involvement were more often associated to KCG. Clinical manifestations are reported in details in Table 1 and presence of clinical manifestations among the two study groups in Fig. 3. ICU admission was more common in KCG (23,1% vs 1,1%; p < 0,0001) as well as the occurrence of secondary Hemophagocytic Lymphohistiocytosis (sHLH) (18,4% vs 1,2%; *p* = 0,001) defined as the presence of HLH-2004 diagnostic criteria [14]. Median length of hospital stay was 11 days (IQR: 8–15 days); KCG presented a longer hospitalization (12 days; IQR: 9–17 days vs 10 days; IQR: 7–14 days; *p* = 0,02). No death was reported.

**Table 1:** Clinical manifestations at disease onset of KawaCOVID Group (KCG) of patients and Kawasaki Disease Group (KDG) of patients

	KCG (***n*** = 53) N (%)	KDG (***n*** = 96) N (%)	***p*** value
**Skin involvement**			
Maculo-papular rash	21 (40)	59 (62)	0**·**001
Erythema multiforme	4 (8)	7 (7)	1
Scarlatiniform rash	0 (0)	8 (8)	0**·**05
Hands and feet erythema/oedema	15 (28)	38 (40)	0**·**17
Hands and feet desquamation	1 (2)	6 (6)	0**·**42
Perineal Erythema/Desquamation	0 (0)	8 (8)	0**·**05
**Mucosal involvement**			
Cheilitis	14 (26)	46 (48)	0**·**01
Pharyngeal and/or oral erhytema	24 (45)	49 (51)	0**·**50
Strawberry tongue	4 (8)	14 (15)	0**·**29
**Ocular involvement**			
Non Secretive Conjunctivitis	27 (51)	67 (70)	0**·**02
**Neurological involvement**			
Headache	5 (9)	3 (3)	0**·**13
Consciousness abnormalities	4 (8)	1 (1)	0**·**05
Aseptic meningitis	3 (6)	1 (1)	0**·**13
Irritability	8 (15)	34 (35)	0**·**01
**Gastrointestinal involvement**			
Diarrhea	28 (53)	11 (12)	< 0**·**0001
Vomiting	14 (26)	8 (8)	0**·**003
Abdominal pain	17 (32)	10 (10)	0**·**001
Gallbladder hydrops	2 (4)	3 (3)	1
**Respiratory involvement**			
Dyspnea	8 (15)	1 (1)	0**·**001
Tachypnea	12 (23)	4 (4)	0**·**001
Cough	3 (6)	10 (10)	0**·**38
Rhinitis	0 (0)	5 (5)	0**·**16
Lobar pneumonia	3 (6)	2 (2)	0**·**35
Interstitial pneumonia	20 (37)	17 (18)	0**·**01
O2 supplementation	14 (28)	2 (2)	< 0**·**0001
Ventilation	6 (12)	0 (0)	0**·**003
**Osteoarticular involvement**			
Arthritis and/or arthralgia	6 (11)	13 (14)	0**·**7

KCG presented, moreover, a lower number of leucocytes, lymphocytes, monocytes and platelets, while CRP was higher in KCG as well as troponin, troponin-T, ferritin and d-dimer (Table 2).

**Table 2:** Laboratory data at disease onset of KawaCOVID Group (KCG) and Kawasaki Disease Group (KDG)

	KCG (***n*** = 53) Median (IQR)	KDG(***n*** = 96) Median (IQR)	***p*** value
**Leucocytes (n/mmc)**	11,500 (7310–15,830)	15,730 (11180–19,810)	0**·**003
**Neutrophils (n/mmc)**	9156 (6480–14,230)	10,617 (6000–14,780)	0**·**5
**Lymphocytes (n/mmc)**	940 (630–1570)	2790 (1680–4340)	<**·**0001
**Monocytes (n°/mmc)**	320 (160–530)	960 (540–1390)	<**·**0001
**Hemoglobin (g/dL)**	11,0 (10,1-11,8)	10,8 (10,0–11,7)	0**·**31
**Platelets (n/mmc)**	186,000 (132000–292,000)	402,500 (242000–523,000)	<**·**0001
**ALT (U/L)**	27 (18–63)	29 (18–81)	1
**AST (U/L)**	36 (20–65)	30 (21–50)	0**·**56
**GGT (U/L)**	31 (16–67)	28 (13–59)	0**·**59
**CRP (mg/L)**	242 (138,5-300,5)	96,6 (40,6–195)	<**·**0001
**IgA (mg/dL)**	134,5 (98–150)	83 (48,5-160,5)	0**·**17
**IgG (mg/dL)**	857 (655–857)	821 (600–1.106)	0**·**89
**IgM (mg/dL)**	90 (75–116)	98,5 (90–152,5)	0**·**31
**Total protein (g/dL)**	6 (5,2-6,7)	6,6 (5,9-7,1)	0**·**03
**Albumin (g/dL)**	2,9 (2,6-3,5)	3,4 (2,8-3,7)	0**·**05
**CPK (U/L)**	57 (32–136)	52 (33–117)	0**·**55
**Troponin-T (ng/L)**	82,5 (20–126)	3,5 (3–4)	0**·**002
**Fibrinogen (mg/dL)**	643 (515–740)	570,5 (468–714)	0**·**14
**aPTT (ratio)**	1,2 (1,0-1,3)	1,1 (1,0-1,2)	0**·**12
**C3 (mg/dL)**	139,5 (138–141)	138 (121–196)	0**·**89
**C4 (mg/dL)**	27,5 (22–33)	36 (24–46)	0**·**89
**Ferritin (ng/mL)**	563,8 (250–1.068)	227 (147–449)	0**·**0004
**D-dimer (ng/mL)**	2514 (1.380–3.890)	1740 (730–2.530)	0**·**03
**NT-BNP (pg/mL)**	927 (701–1.734)	347 (81–350)	0**·**08
**ESR (mm/hr)**	59 (36–81)	66,5 (49,5–98)	0**·**18
**aPTT (sec)**	31,7 (30,9-32,4)	26,9 (23–30)	0**·**16
**PT (sec)**	14,3 (13,2-15,2)	75,5 (55–88)	0**·**08
**Troponin (ng/L)**	168 (80–672)	4 (3–24)	0**·**001
**Triglycerides (mg/dL)**	217,5 (137–261)	144,5 (122–196)	0**·**04

### Cardiac involvement

Seventy-seven patients showed heart involvement during the course of the disease: 35 had myocarditis (60,4% KCG vs 3,1% KDG; *p* < 0,0001), 21 pericarditis (26,4% KCG vs 7,3 KDG; p = 0,0013), 19 valvular insufficiency (24,5% KCG vs 6,3% KDG; p = 0,0036), 34 coronary artery abnormalities (CAA) (13,2% KCG vs 28,1% KDG; p = 0,0427), 20 heart failure (35,8% KCG vs 1% KDG; *p* < 0,00001) and 21 hypotension/non-cardiogenic shock (37,8% KCG vs 1% KDG; *p* < 0,0001).

Data on heart ultrasonography at follow-up was available for 138 patients out of 149. Median time at follow/up was 39,9 days (SD ± 20,79) after discharge. Normal echocardiograms were showed by 77,2% of patients; 15,4% showed persistent heart ultrasonography abnormalities (15,7% KCG vs 17,2% KDG; *p* = 1); in details, 6 out of 8 (75%) KCG still presented persistent mild heart failure and 2 out of 8 (25%) patients still had valvular insufficiency; among KDG, 10 out of 15 (66,7%) children had minimal CAA, 1 patient showed mild pericarditis, 1 patient heart failure and 3 had valvular insufficiency.

### Treatment

One-hundred-twenty-eight patients received intravenous immunoglobulins (IVIG), 69 (53,9%) glucocorticoids (67 out of them received intravenous glucocorticoids), 123 (99,9%) acetylsalicylic acid (ASA), 4 (3,1%) hydroxychloroquine, 9 (7%) patients anakinra and 1 (0,8%) tocilizumab, 6 (4,7%) anti-viral agents, 93 (72,7%) antibiotics, 16 (12,5%) vasoactive agents and 27 (21,1%) heparins. At time of diagnosis IVIG and ASA were used more frequently in KDG (81,3% vs 66%; *p* = 0,04 and 71,9% vs 43,4%; p = 0,001 respectively) while glucocorticoids were more often used in patients with KCG (56,6% vs 14,6%; *p* < 0,0001). Three patients with KCG received anti-IL1 treatment (anakinra) and 1 patient hydroxychloroquine. Antiviral treatment was used only in three patients (2 in the KCG and 1 in KDG), while antibiotics, vasoactive drugs and heparin were used more frequently in KCG (73,6% vs 35,4%, *p* < 0,0001; 20,8% vs 0,0%; p < 0,0001 and 28,3% vs 1,0%; p < 0,0001 respectively).

Among KCG the use of IVIG alone, glucocorticoids alone or combined with IVIG at the time of diagnosis was not associated with a higher incidence of clinical worsening (Additional file 1) and/or presence of cardiac involvement at follow-up (16/106 vs 8/34; *p* = 0,1).

### Relationship between SARS-CoV-2 infection and KawaCOVID

One-hundred-thirty-six patients out of 149 were tested for SARS-CoV-2 with RT-PCR assay and/or with serologic assay. Fifty-four patients resulted positive (14 only for RT-PCR, 31 only for serologic assay and 9 for both). The positive assays for SARS-CoV-2 were higher in KCG (75,5% vs 20%; p < 0·0001).

We compared the data of patients from two regions with higher SARS-CoV-2 infection (Piedmont and Lombardy) (76 patients) (Group A) with the data of patients from the other regions (73 patients) (Group B). Group A patients presented more often with diarrhea (36,8% vs 15,1%; *p* = 0,003), myocarditis (29,0% vs 9,6%; p = 0,003), pericarditis (15,8% vs 2,7%; *p* = 0,01) and hypotension/non-cardiogenic shock (17,1% vs 2,7%; p = 0,01). Again, Group A patients were confirmed to be significantly older (4 years; IQR: 2–8 years vs 2,5 years; IQR: 1–4 years; p = 0,004). Laboratory test showed lower WBC (11,700/mm3; IQR: 7960–16,900/mm3 vs 16,250/mm3; IQR: 11180–19,810/mm3; p = 0,03), lymphocytes (1390/mm3; IQR: 790–2570/mm3 vs 2562,5/mm3; IQR: 1515–4255/mm3; p = 0,0003) and platelets (225,000/mm3; IQR: 148000–389,000/mm3 vs 361,000/mm3; IQR: 244000–471,000/mm3; p = 0,01) in group A. Patients from group A also had higher levels of CRP (195 mg/L; IQR: 82,2–266,7 mg/L vs 96,6 mg/L; IQR: 40,6–197,9 mg/L; p = 0,003), and higher levels of ferritin (449 ng/mL; IQR: 227–745 ng/mL vs 218 ng/mL; IQR: 145–458 ng/mL; *p* = 0,02).

**Fig. 4:**
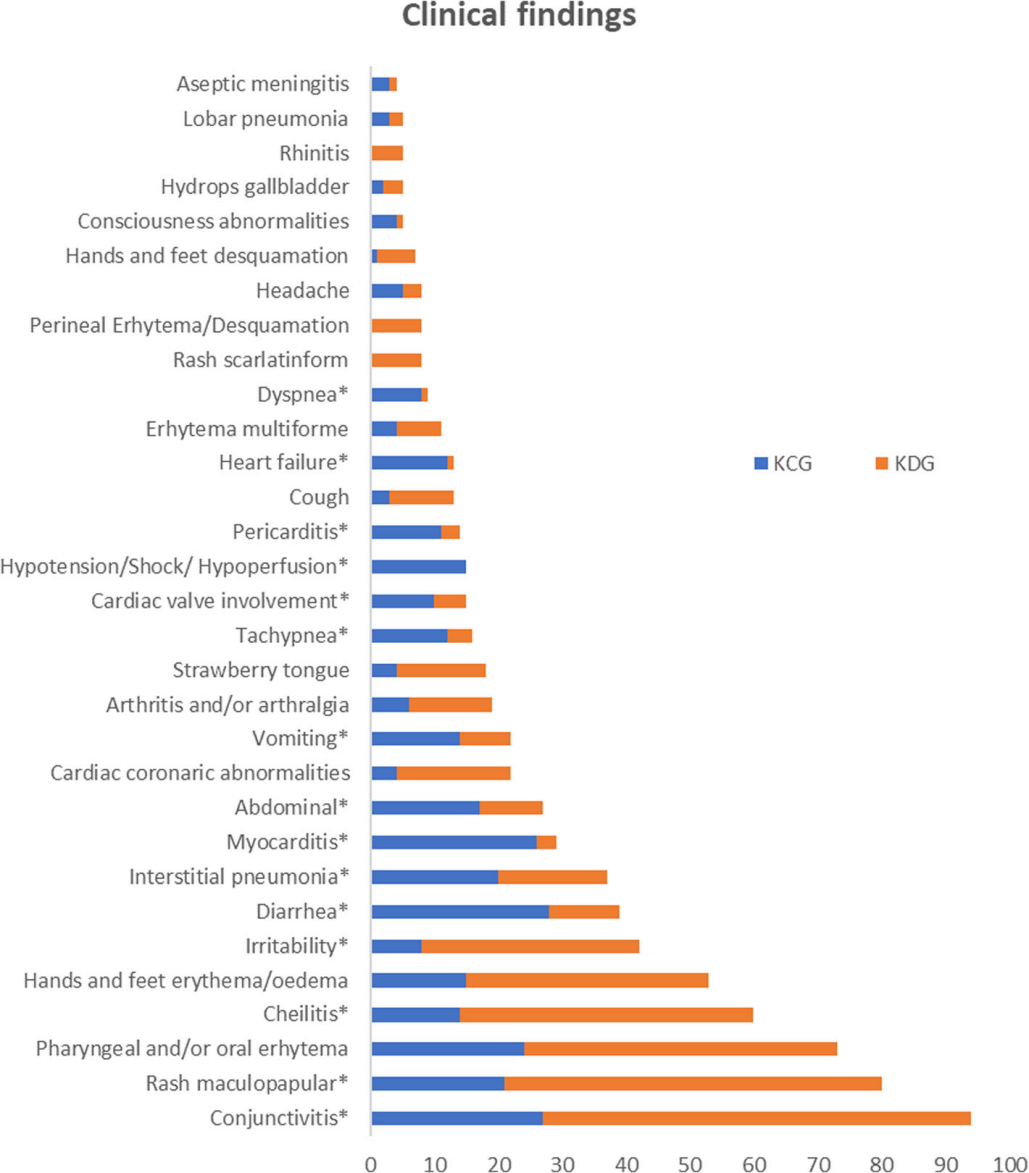
Relationship between SARS-CoV-2 infection trend and new cases of Kawasaki Disease and KawaCOVID during SARS-CoV-2 infection in Italy from February 1st 2020 to May 31st 2020

While the KDG cases were spread during the study period, the KCG cases were mainly concentrated towards the end of the observation period with about 1 month of delay compared to the peak of the SARS-CoV-2 epidemic (Fig. 4).

### Kawasaki disease during SARS-CoV-2 epidemic

Clinical characteristics of KDG during SARS-CoV-2 epidemic (KDG-CoV2) were compared to 598 Kawasaki Disease patients (KDG-Historical) seen in three different Italian tertiary pediatric hospitals (Institute for Maternal and Child Health, IRCCS “Burlo Garofolo”, Trieste; AOU Meyer, Florence; IRCCS Istituto Giannina Gaslini, Genoa) from January 1st 2000 to December 31st 2019.

No difference was found in age at time of diagnosis, Female/Male ratio, classical/incomplete Kawasaki Disease and clinical manifestations (conjunctivitis, lymphoadenopathy, cheilitis, skin rash, extremities changes, sterile pyuria, gastrointestinal involvement, respiratory symptoms, musculoskeletal symptoms, neurological symptoms, prevalence of CAA, myocarditis, pericarditis, and valvular insufficiency) between KDG and KD-Historical group respectively. No difference was also found in laboratory data available (Additional files 2 and 3). KDG patients from highly endemic areas (Piedmont and Lombardy) were compared to those of medium-low endemic areas. No significant statistical difference was found in clinical manifestations and laboratory data between the two groups (Additional files 4 and 5).

## Discussion

The occurrence of a severe pediatric inflammatory disease, with some characteristics of KD, has been described since late march, in areas with high SARS-CoV-2 incidence [5, 7, 9, 15–18]. Whether this is a particularly aggressive form of KD triggered by SARS-CoV-2 or a completely different entity is still a matter of debate.

Although some case series have already been reported [15–18], our national survey was aimed to collect all patients affected by KD and KD-like inflammatory symptoms during SARS-CoV-2 epidemic.

Notably, after the first cases of SARS-CoV-2 in the northern Italy regions, the lock-down policy reduced the spread of the infection through other regions.

We confirmed some clinical evidence already reported in the literature; KCG patients are older at disease onset and present more frequently gastrointestinal and respiratory symptoms [3], while the classic mucocutaneous symptoms of KD were less common. Children in the KCG also showed higher markers of inflammation, with lower WBC, and platelets [7, 8]. Indeed, lymphopenia is very common in patients with SARS-CoV-2. Accordingly, patients from the KCG were at higher risk of developing sHLH which is indeed rarely described in children with KD [19].

Other authors compared patients with PIMS-TS or analogs with a historic cohort of KD patients obtaining similar results [7, 9, 16]; the importance of our observation relies on the fact that out KDG was recruited during the same period of KCG with a controlled patients selection.

Patients of the KCG did also require longer time in the hospital and needed more frequently ICU admittance, for the occurrence of shock, need of vasoactive agents, and invasive ventilation. In the UK, it has been recently reported a higher incidence of ICU admittance of patients with unexplained inflammatory conditions suggestive of PIMS-TS where 2 out of 78 children died [20]. No death was reported in our cohort. Although we did not find any significant correlation between initial treatment and outcome at the end of follow-up, it is important to underline that the use of glucocorticoids was more frequently reported among the KCG and that all patients among KCG received glucocorticoids, IVIG or both within 24 h since admission. This is to confirm that maybe a more aggressive treatment at the time of admission might prevent clinical complications or even death. We would suggest, in absence of stronger clinical evidence, that early treatment with both IVIG (2 g/kg/day) and glucocorticoids (methylprednisone 2 mg/kg/day) is indicated in KawaCOVID patients; in our experience treatment with parenteral anakinra (from 2 mg/kg/day to 10 mg/kg/day) is indicated in those patients with rapid clinical worsening or if clinical improvement is not reached within 48 h since starting IVIG and corticosteroids. Our hypothesis seems to be confirmed from Navallo-Millan et al., suggesting that anakinra could be beneficial in COVID-19 adult patients with evidence of cytokine storm syndrome when initiated early after the onset of Acute Hypoxic Respiratory Failure [21].

Heart involvement is peculiar to both KDG and KCG [6, 22]. The major complication in KDG patients was the occurrence of CAA while KCG patients were at higher risk to develop myocarditis with heart insufficiency and related manifestations, such as valvular insufficiency. One of the main unanswered questions on PIMS-TS/MIS-C is if patients with this disease are at risk of developing CAA in the follow/up, as described for KD. For the first time we were able to provide short-term follow-up data on our KCG patients, showing that a minority of patients had minor complications, sequelae of the acute myocarditis, while the 3 KCG patients that developed CAA during hospital stay (aneurysms in 2 patients, dilation in 1), had resolution of the coronary involvement at follow-up. This was in contrast with data from the KDG, where 10/15 patients still presented at follow/up with CAA.

Although PIMS-TS and analogs are considered a complication of SARS-CoV-2 infection, the results from nasal swabs PCR and serologies are usually variable [14, 23]. Data on SARS-CoV-2 exposure were present in 136/149 patients from our cohort. Evidence of SARS-CoV-2 infection, with limit related to the availability of the text at time of the study, was more frequent in patients from the KCG. As already mentioned, SARS-CoV-2 spread with different incidence through Italy. When comparing patients from high endemic regions vs patients from low endemic ones, results were very similar to the analysis of KCG vs KDG, confirming the hypothesis that KawaCOVID is strictly related to SARS-CoV-2 infection.

Similar to other reports, there was a 4-weeks delay in our population between the number of patients with COVID-19 in the general population and the peak of KawaCOVID [9]. Very interestingly, while the number of KawaCOVID cases in the observation period showed the pattern already described, the number of KD was constant throughout the same period. KD incidence is estimated as 5,7 per 100,000 children 0–14 y/o in Italy [24]. Considering only KD diagnosis, the estimated incidence in our study was 3,99 per 100,000 children < 14y/o, 5,48/100000 children < 14 y/o considering only patients from highly endemic regions (Piedmont and Lombardy). Notably, since participation in our registry was voluntary, cases may have been under-reported.

Since some of our patients with KD had evidence of SARS-CoV-2 infection, we compared patients from KDG with a historic cohort of Italian KD patients to better define if SARS-CoV-2 could be a trigger of KD and determine a specific pattern of disease. There was no difference in clinical or laboratory characteristics and outcome between the two populations, suggesting that, if SARS-CoV-2 could trigger KD, it does not determine specific disease features.

There is still a debate whether KawaCOVID may be considered a separate clinical entity or a more severe form of KD [25], specifically triggered by SARS-CoV-2. The stronger evidence suggesting that PIMS-TS is an severe manifestation of Kawasaki Disease comes from the remarkable overlapping of many clinical features, mainly the mucocutaneous manifestations and the possible formation of coronary artery aneurysms. This overlapping is even more obvious when considering a rare subtype of KD, described by Kanegaye *et al* [26] and called Kawasaki Shock Syndrome (KSS), whose main feature is the occurrence of shock. Another important point towards this hypothesis is the good treatment response to systemic glucocorticoids and IVIG [27].

On the other hand, previous data suggested that patients with PIMS-TS have some peculiar characteristics when compared to classic KD patients or even KSS [7, 9]. We suggest that SARS-CoV-2 might determine two types of inflammatory diseases in children: the first manifestation is the classic KD, that could be triggered by the coronavirus, as already suggested [28]. According to our results, the occurrence of this SARS-CoV-2-triggered KD is rare and does not impact significantly on the annual incidence of the disease. The second manifestation related to SARS-CoV-2 exposure during childhood is the multisystem inflammatory syndrome, which affects older children and presents mainly with myocarditis, gastrointestinal symptoms, and the occurrence of shock. Since it appears this latter disease is distinct from KD we recognize the term KawaCOVID could be misleading and both PIMS-TS or MIS-C seem more appropriate. Age at SARS-CoV-2 infection, previous coronaviruses exposure [22], host immunity, and predisposing genetics might have a role in determining which patients would develop a SARS-CoV-2 related disease.

Even though PIMS-TS/MIS-C is a severe disease, the majority of patients respond to treatments, with few complications and a good outcome. As prospective data on larger series are not available, our experience suggests a prompt treatment with glucocorticoids and IVIG. The use of anakinra is also to take into account if clinical worsening is present or in case of a lack of clinical response. Ongoing studies on the treatment of inflammatory conditions related to SARS-CoV-2 might help to define the best options for PIMS-TS patients [29].

## Conclusions

Our study suggests that SARS-CoV-2 infection is the causative agent of PIMS-TS in children. Older age at onset and clinical peculiarities like the occurrence of myocarditis characterize this multi-inflammatory syndrome which share specific clinical manifestations with KD. Our patients had an excellent response to treatment and a good outcome, with few complications and no deaths.

## Supplementary Information

**Additional file 1: Appendix 1.**

**Additional file 2: Appendix 2.** Clinical comparison between Kawasaki Disease patients seen during SARS-CoV-2 epidemic and a Historical Cohort of Kawasaki Disease Patients.

**Additional file 3: Appendix 3.** Laboratory comparison between Kawasaki Disease patients seen during SARS-CoV-2 epidemic and a Historical Cohort of Kawasaki Disease Patients.

**Additional file 4: Appendix 4.** Clinical comparison between Kawasaki Disease patients seen during SARS-CoV-2 in high epidemic regions (Piedmont and Lombardy) and Kawasaki Disease Patients in low epidemic regions.

**Additional file 5: Appendix 5.** Comparison of laboratory tests between Kawasaki Disease patients seen during SARS-CoV-2 in high epidemic regions (Piedmont and Lombardy) and Kawasaki Disease Patients in low epidemic regions.

## Data Availability

The datasets used and/or analysed during the current study are available from the corresponding author on reasonable request.

## Abbreviations

ASA: Acetylsalicylic acid
CAA: Coronary artery abnormalities
CRP: C-Reactive Protein
iKD: Incomplete Kawasaki Disease
IVIG: Intravenous immunoglobulins
MIS-C: Multisystem Inflammatory Syndrome associated with Coronavirus Disease
KCG: KawaCovid Group
KDG: Kawasaki Disease Group
PIMS-TS: Pediatric Inflammatory Multisystem Syndrome-temporally associated to SARS-CoV-2 infection
RT-PCR: Real Time-Polimerase Chain Reaction
sHLH: Secondary Hemophagocytic Lymphohistiocytosis
TSS: Toxic Shock Syndrome
