# Supplementary material for: Defining Kawasaki disease and pediatric inflammatory multisystem syndrome-temporally associated to SARS-CoV-2 infection during SARS-CoV-2 epidemic in Italy: results from a national, multicenter survey
Source: Pediatr Rheumatol Online J. 2021 Mar 16;19:29. doi: 10.1186/s12969-021-00511-7 (PMC7962084; doi:10.1186/s12969-021-00511-7)
Supplement: Supplementary file 1 — Additional file 1: Appendix 1. [file 12969_2021_511_MOESM1_ESM.docx]

**Appendix 1.**

|  | **Clinical worsening during follow-up** | | |
| --- | --- | --- | --- |
|  | YES | NO | p |
| **IVIG at time of diagnosis** |  |  | 0.38 |
| YES | 41 | 72 |  |
| NO | 16 | 20 |  |
| **Corticosteroids at time of diagnosis** |  |  | 0.5 |
| YES  NO | 15  42 | 29  63 |  |
| **Corticosteroids plus IVIG at time of diagnosis** |  |  | 0.23 |
| YES  NO | 68  24 | 47 10 |  |
