# Supplementary material for: Defining Kawasaki disease and pediatric inflammatory multisystem syndrome-temporally associated to SARS-CoV-2 infection during SARS-CoV-2 epidemic in Italy: results from a national, multicenter survey
Source: Pediatr Rheumatol Online J. 2021 Mar 16;19:29. doi: 10.1186/s12969-021-00511-7 (PMC7962084; doi:10.1186/s12969-021-00511-7)
Supplement: Supplementary file 2 — Additional file 2: Appendix 2. Clinical comparison between Kawasaki Disease patients seen during SARS-CoV-2 epidemic and a Historical Cohort of Kawasaki Disease Patients. [file 12969_2021_511_MOESM2_ESM.docx]

|  | **Kawasaki Disease Group** | **Kawasaki Historical Group (%)** | **p** |  |
| --- | --- | --- | --- | --- |
| **Male Gender (%)** | 45,8 | 40 | 0,29 | |
| **Atypical or Incomplete Kawasaki (%)** | 35,4% | 34,3% | 0,83 | |
| **Conjunctivits (%)** | 83,3 | 81,2 | 0,62 | |
| **Lymphoadenopathy (%)** | 61,5 | 52,8 | 0,12 | |
| **Cheilitis (%)** | 85,4 | 81 | 0,30 | |
| **Rash (%)** | 87,5 | 86 | 0,69 | |
| **Extremities changes** | 51 | 53,7 | 0,65 | |
| **Gastrointestinal involvement** | 32,3 | 41,6 | 0,11 | |
| **Respiratory symptoms** | 19,8 | 25,2 | 0,29 | |
| **Musculoskeletal isymptoms** | 17,7 | 21,4 | 0,44 | |
| **Neurological involvement** | 43,7 | 47,9 | 0,49 | |
| **Sterile pyuria** | 14,6 | 10 | 0,13 | |
| **Coronary Artery Abnormalities** | 28,1 | 25,4 | 0,57 | |
| **Myocarditis** | 4,2 | 6 | 0,6 | |
| **Pericarditis** | 7,3 | 9,2 | 0,84 | |
| **Valvular Insufficiency** | 5,2 | 7,8 | 0,52 | |

Appendix 2.

Clinical comparison between Kawasaki Disease patients seen during SARS-CoV-2 epidemic and a Historical Cohort of Kawasaki Disease Patients.
