# Supplementary material for: Defining Kawasaki disease and pediatric inflammatory multisystem syndrome-temporally associated to SARS-CoV-2 infection during SARS-CoV-2 epidemic in Italy: results from a national, multicenter survey
Source: Pediatr Rheumatol Online J. 2021 Mar 16;19:29. doi: 10.1186/s12969-021-00511-7 (PMC7962084; doi:10.1186/s12969-021-00511-7)
Supplement: Supplementary file 3 — Additional file 3: Appendix 3. Laboratory comparison between Kawasaki Disease patients seen during SARS-CoV-2 epidemic and a Historical Cohort of Kawasaki Disease Patients. [file 12969_2021_511_MOESM3_ESM.docx]

|  | **Kawasaki Disease Group**  **Median (SD)** | **Historical Kawasaki Disease Group**  **Median (SD)** | ***p* value** |
| --- | --- | --- | --- |
| **Neutrophils (n/mmc)** | 11217 (6571) | 11372 (5335) | 0,48 |
| **Lymphocytes (n/mmc)** | 3084 (1887) | 3298 (2164) | 0,56 |
| **Hemoglobin (g/dL)** | 10,9 (1,2) | 11,2 (5,4) | 0,81 |
| **Platelets (n/mmc)** | 420979 (238310) | 426586 (179880) | 0,25 |
| **ALT (U/L)** | 67 (63) | 76 (55) | 0,76 |
| **AST (U/L)** | 66 (42) | 67 (44) | 0,3 |
| **GGT (U/L)** | 47 (41) | 75 (66) | 0,1 |
| **CRP (mg/L)** | 11,5 (8,7) | 10,9 (10,4) | 0,4 |
| **Ferritin (ng/mL)** | 381 (243) | 299 (212) | 0,12 |
| **ESR (mm/hr)** | 72 (30) | 73 (30) | 0,55 |

Appendix 3. Laboratory comparison between Kawasaki Disease patients seen during SARS-CoV-2 epidemic and a Historical Cohort of Kawasaki Disease Patients.
