# Supplementary material for: Defining Kawasaki disease and pediatric inflammatory multisystem syndrome-temporally associated to SARS-CoV-2 infection during SARS-CoV-2 epidemic in Italy: results from a national, multicenter survey
Source: Pediatr Rheumatol Online J. 2021 Mar 16;19:29. doi: 10.1186/s12969-021-00511-7 (PMC7962084; doi:10.1186/s12969-021-00511-7)
Supplement: Supplementary file 4 — Additional file 4: Appendix 4. Clinical comparison between Kawasaki Disease patients seen during SARS-CoV-2 in high epidemic regions (Piedmont and Lombardy) and Kawasaki Disease Patients in low epidemic regions. [file 12969_2021_511_MOESM4_ESM.docx]

|  | **Kawasaki Disease Piedmont/Lombardy** | **Kawasaki Disease**  **Other regions** | **p** |
| --- | --- | --- | --- |
| **Age to onset (year; SD)** | 3 (3) | 2,4 (2,1) | 0,74 |
| **Conjunctivits (%)** | 65,7 | 72,1 | 0,51 |
| **Lymphoadenopathy (%)** | 56,2 | 24 | 0,003 |
| **Cheilitis (%)** | 37,1 | 54,1 | 0,11 |
| **Rash (%)** | 97,14 | 93,44 | 0,65 |
| **Extremities changes** | 40 | 39,3 | 0,95 |
| **Gastrointestinal involvement** | 45,7 | 42,6 | 0,77 |
| **Respiratory symptoms** | 71,4 | 54,1 | 0,09 |
| **Musculoskeletal isymptoms** | 22,8 | 34,4 | 0,23 |
| **Neurological involvement** | 48,6 | 57,3 | 0,4 |
| **Sterile pyuria** | 5,7 | 9,8 | 0,71 |
| **Coronary Artery Abnormalities** | 22,9 | 16,4 | 0,72 |
| **Myocarditis** | 8,6 | 0 | 0,05 |
| **Pericarditis** | 5,7 | 1,6 | 0,55 |
| **Valvular Insufficiency** | 5,7 | 4,9 | 1 |

Appendix 4.

Clinical comparison between Kawasaki Disease patients seen during SARS-CoV-2 in high epidemic regions (Piedmont and Lombardy) and Kawasaki Disease Patients in low epidemic regions.
