# Supplementary material for: Defining Kawasaki disease and pediatric inflammatory multisystem syndrome-temporally associated to SARS-CoV-2 infection during SARS-CoV-2 epidemic in Italy: results from a national, multicenter survey
Source: Pediatr Rheumatol Online J. 2021 Mar 16;19:29. doi: 10.1186/s12969-021-00511-7 (PMC7962084; doi:10.1186/s12969-021-00511-7)
Supplement: Supplementary file 5 — Additional file 5: Appendix 5. Comparison of laboratory tests between Kawasaki Disease patients seen during SARS-CoV-2 in high epidemic regions (Piedmont and Lombardy) and Kawasaki Disease Patients in low epidemic regions. [file 12969_2021_511_MOESM5_ESM.docx]

|  | **Kawasaki Disease high eidemic area(n=36)**  **Median (SD)** | **Kawasaki Disease Patients low epidemic area (n=90)**  **Median (SD)** | ***p* value** |
| --- | --- | --- | --- |
| **Leucocytes (n/mmc)** | 14230 (7363) | 16949 (7421) | 0**·**08 |
| **Neutrophils (n/mmc)** | 9522 (5421) | 3521 (2066) | 0**·**13 |
| **Lymphocytes (n/mmc)** | 2704 (1740) | 2790 (1680-4340) | 0**·**06 |
| **Monocytes (n°/mmc)** | 890 (516) | 1020 (524) | 0·27 |
| **Hemoglobin (g/dL)** | 10,6 (1,34) | 10,9 (0,99) | 0**·**21 |
| **Platelets (n/mmc)** | 391935 (233389) | 449638 (255300) | 0**·**34 |
| **ALT (U/L)** | 77 (129) | 69 (84) | 0,92 |
| **AST (U/L)** | 81 (164) | 63 (125) | 0**·**78 |
| **GGT (U/L)** | 55 (62) | 46 (47) | 0**·**95 |
| **CRP (mg/L)** | 152 (101) | 99 (81) | 0**·**04 |
| **Total protein (g/dL)** | 6,3 (0,69) | 6,6 (0,95) | 0**·**22 |
| **Albumin (g/dL)** | 3,1 (2,6-3,5) | 3,7 (2,8-3,7) | 0**·**3 |
| **aPTT (ratio)** | 1,09 (0,12) | 2 (4,4) | 0**·**64 |
| **Ferritin (ng/mL)** | 360 (201) | 457 (1202) | 0**·**03 |
| **D-dimer (ng/mL)** | 2241 (1244) | 1356 (1189) | 0**·**11 |
| **ESR (mm/hr)** | 68 (27) | 72 (32) | 0**·**99 |
| **Troponin (ng/L)** | 22,7 (39,7) | 34,6 (54) | 1 |

Appendix 5. Comparison of laboratory tests between Kawasaki Disease patients seen during SARS-CoV-2 in high epidemic regions (Piedmont and Lombardy) and Kawasaki Disease Patients in low epidemic regions.
